# Moderate-to-vigorous and light-intensity aerobic exercise yield similar effects on food reward, appetitive responses, and energy intake in physically inactive adults

**DOI:** 10.1038/s41430-025-01574-5

**Published:** 2025-02-28

**Authors:** Shu-Shih Hsieh, Angelos Bala, Kapris Layzell, Qanita Fatima, Clarciya Pushparajah, Rebecca K. Maguire, Yung-Chih Chen, Graham Finlayson, Judith E. Allgrove

**Affiliations:** 1 Department of Psychology, Kingston University London, Kingston upon Thames, UK; 2 Department of Physical Education and Sport Sciences, National Taiwan Normal University, Taipei, Taiwan; 3 School of Psychology, Faculty of Medicine and Health, University of Leeds, Leeds, UK; 4 Department of Applied and Human Sciences, Kingston University London, Kingston upon Thames, UK; 5 Present Address: Department of Rehabilitation and Sport Sciences, Bournemouth University, Poole, UK

**Keywords:** Nutrition, Lifestyle modification

## Abstract

**Objective:**

To examine the effect of acute aerobic exercise at moderate-to-vigorous and light intensity on food reward, appetite sensation, and energy intake (EI) in physically inactive adults.

**Methods:**

Twenty inactive, healthy adults (mean age: 21 ± 3 years) completed two trials (i.e. moderate-to-vigorous and light-intensity exercise) in a randomised, crossover design. Participants performed a 40-min cycling bout at either 50% or 20% peak power output in a counterbalanced order. Before and after exercise bouts, liking and wanting for fat and sweet/savoury categories of food were assessed by the Leeds Food Preference Questionnaire, along with subjective ratings of appetite and state food cravings. EI was evaluated by *ad libitum* meals following exercise bouts.

**Results:**

Results showed increased implicit wanting for high-fat relative to low-fat foods (*p* = .04, d_rm_ = 0.50) and savoury relative to sweet foods following exercise bouts (*p* = .04, d_rm_ = −0.49) without intensity-specific effects. Exercise bouts also led to increased subjective appetite (*p* < 0.001, *η*^2^_p_ = 0.60) and state food cravings (*p* = 0.001, *η*^2^_p_ = 0.44) without intensity-specific differences. There was no difference between exercise intensities on absolute EI, whereas relative EI was lower after moderate-to-vigorous exercise relative to light exercise (*p* < 0.001, d_rm_ = −1.56).

**Conclusion:**

Acute aerobic exercise, regardless of intensity, may lead to increased wanting for high-fat relative to low-fat and savoury relative to sweet-tasting foods, as well as enhanced appetite sensation and food cravings in physically inactive adults. Moderate-to-vigorous exercise does not induce additional energy intake relative to light exercise.

## Introduction

A growing body of research highlights the need to investigate the hedonic aspect of eating. Unlike homoeostatic eating regulated by energy balance, hedonic eating is driven by behaviours linked to reward and motivational neurocircuitry [1–3]. Dysregulation in non-homoeostatic eating has been associated with psychopathological and metabolic conditions, including eating disorders, food addiction, and obesity [4–6]. Hence, it is relevant to investigate how food reward processes, such as wanting (i.e. the motivation towards certain foods) [7] and liking (i.e. the degree of sensory pleasure obtained from foods) [7] of specific foods, respond to acute bouts of exercise because such investigations may provide an important implication for the prevention and management of hedonic and disordered eating behaviours.

Regarding the effects of acute bouts of exercise on food reward processes, the current findings are equivocal. For instance, whereas 3 studies found decreased implicit (e.g. automatic), and explicit (e.g. conscious) wanting for high-fat foods following acute bouts of aerobic [8–10] and resistance exercise [9], others found that neither liking nor wanting of high-fat foods were changed following acute bouts of aerobic exercise (e.g. cycling) or eccentric exercise (e.g. downhill running) [11, 12]. Similarly, findings regarding the effects of acute exercise on high-sweet foods are inconsistent. Whereas one recent study found increased implicit wanting and preference for high-sweet foods following acute bouts of aerobic exercise [8], 4 studies found a null effect of acute exercise [9–12] These discrepancies could be partly explained by heterogeneities in feeding state of participants (e.g. participants are offered standardised meals versus are fasted upon arrival) and sex distribution of participants (e.g. participants are all men versus are both men and women). Further investigation into the effects of acute exercise on food reward under the same feeding state and in a sample consisting of both men and women is warranted.

Furthermore, two issues remain underexplored in this line of research. First, the converging data on acute exercise-induced appetitive changes are based on individuals who were either recreational exercisers or higher-fit individuals [8–13], leaving acute exercise-induced changes in appetite, food reward, and energy intake underexplored in physically inactive and lower-fit individuals. Further, whereas one recent study found that breaking up sitting with brief bouts of aerobic exercise (e.g. 2-min walking every 20 min over 5.5 h) may not alter post-exercise eating behaviour in physically inactive individuals [14], the effect of continuous aerobic exercise remains unclear. To provide better-personalised exercise recommendations, it is relevant to investigate the abovementioned causal relations in physically inactive individuals. Second, the effects of light-intensity exercise (e.g. slow walking or cycling) on eating behaviours remains unclear. From a public health standpoint, it might be required to investigate the effects of light-intensity exercise on food reward and appetitive responses because this intensity of exercise may be better tolerated and safer for individuals who are physically inactive or lower-fit. Further, a systematic review indicated that frequent short bouts of light-intensity activity improve glycaemic control and cardiometabolic biomarkers (e.g. blood pressure, postprandial glucose), suggesting the relevance of considering acute bouts of light-intensity exercise during the day [15].

Taken together, the objective of the current study was to investigate the differential effects between moderate-to-vigorous and light-intensity exercise on food reward processes, appetite sensation, and energy intake in physically inactive individuals.

## Methods

Twenty-five young adults between 19 and 40 years old were recruited (participants’ characteristics are shown in Table 1**)**. Participants were eligible if they: (1) were classified as physically inactive, which was quantified as engaging in <150 min of moderate-to-vigorous physical activity in the past 7 days [16]. Moderate-to-vigorous physical activity was defined by examples provided by the physical activity guidelines for adults between 19 and 64 years old from the National Health Service, UK, (2) free from any of the medical conditions listed on a Physical Activity Readiness Questionnaire (PAR-Q) [17]; (3) free from cardiovascular, cerebrovascular, neurological, or mental disorders (e.g. depression, anxiety, eating disorders), (4) were not on a restrictive diet or caloric restriction plan, (5) were not taking any hormone-stimulating supplements or medication, such as oral contraceptive, and (6) were not vegan or vegetarian. The study protocol was conducted in accordance with the declaration of Helsinki and was approved by the ethics review committee at Kingston University (MR-2989). Five participants withdrew from the study due to personal reasons or incompliance with study procedures. The data reported herein were, therefore, based on 20 participants (see Supplementary 1 for statistical power estimation).

**Table 1:** Demographic statistics of participants (Mean ± SD).

Variable	Men (*n* = 10)	Women (*n* = 10)	Overall
Age (years)	20.8 (2.9)	21.1 (3.1)	21.0 (2.9)
BMI (kg/m^2^)	21.2 (3.7)	23.3 (2.0)	22.2 (3.1)
PPO (watts)	189.9 (50.2)	138.1 (15.2)	164.0 (44.8)
Estimated VO_2max_ (ml kg min^−1^)	39.1 (7.4)	32.8 (5.1)	36.0 (7.0)
EDE (AU)			
Global score	0.3 (0.4)	0.8 (1.0)	0.6 (0.8)
Restraint eating	0.4 (0.6)	0.9 (1.2)	0.6 (1.0)
Eating concern	0.0 (0.1)	0.4 (0.4)	0.2 (0.3)
Shape concern	0.5 (0.7)	1.0 (1.3)	0.8 (1.1)
Weight concern	0.3 (0.7)	1.0 (1.4)	0.7 (1.1)
TFEQ (AU)			
Cognitive restraint	12.8 (1.5)	12.2 (1.8)	12.5 (1.6)
Uncontrolled eating	24.8 (2.7)	20.7 (3.5)	22.8 (3.7)
Emotional eating	11.2 (0.9)	8.3 (2.5)	9.8 (2.3)
FCQ-T (AU)	22.8 (7.5)	37.4 (11.4)	30.1 (12.0)
CESD-10 (AU)	6.5 (4.4)	8.6 (4.4)	7.6 (4.4)

*BMI* body mass index, *PPO* peak power output, *EDE* eating disorder questionnaire, *TFEQ* three-factor eating questionnaire, FCQ-T food craving questionnaire- Trait, *CESD* Centre for Epidemiological Studies Depression Scale, AU arbitrary unit.

Before the first main trial, participants visited the laboratory to undergo preliminary assessments and to be familiarised with the laboratory environment, the study procedures, and materials of the Leeds Food Preference Questionnaire (LFPQ). Upon arrival, participants provided informed consent and completed questionnaires collecting demographic data (see Table 1 for details). Afterward, participants completed a series of questionnaires assessing their eating behaviour traits and mental health (see Supplementary 1 for details of questionnaires and Table 1 for results). During this visit, participants also confirmed acceptability of the *ad libitum* meals that were provided during the two main trials. At the end of the preliminary visit, participants underwent the Peak Power Output (PPO) test (see Supplementary 1 for protocol). The preliminary visit took 1 h to complete.

The first main trial was arranged at least 48 h after the preliminary trial. For the main trials, there was at least 1-week interval between trials (mean interval between trials: 16 ± 11 days) to washout practice and carryover effects. Participants visited the laboratory at the same day of the week and same time of the day. Before each main trial, participants were given instructions to abstain from structured physical activity, caffeine, nicotine, and alcohol for 24 h, and fast for at least 4 h (only water was allowed). Participants were also told to take record of their last two meals prior to the first main trial and were asked to follow the same diets before the second main trial. To minimise the confounding effects of menstrual cycle, female participants only visited the laboratory during either the early-to-mid follicular phase or the mid-luteal phase (the same participants visited the laboratory during the same phase). All these pre-visit requirements were confirmed upon participants’ arrival.

Each main trial lasted 2.5 h. To increase the ecological validity of research, we had participants visit the laboratory at either 10 a.m., 3 p.m., or 4 p.m. so that the *ad libitum* meals were always taken at regular lunch (e.g. between 12 and 12:30 p.m. for 10 a.m. visit) or dinner times (e.g. between 5 p.m. and 5:30 p.m. for 3 p.m. visit or between 6 p.m. and 6:30 p.m. for 4 p.m. visit). Upon arrival, participants sat in a neuropsychology testing room and were fitted with a heart rate (HR) monitor (H10, Polar Electro Oy, Finland). Next, participants sat quietly in the testing room for 5 min to measure their resting HR. Afterward, participants completed appetite ratings (appetite visual analogue scale [VAS] for fullness, hunger, desire to eat, and prospective food consumption) [18, 19], food craving questionnaire-state (FCQ-S) [20], and LFPQ [21, 22] as pre-test assessments (see Supplement 1 for details of appetite ratings, FCQ-S, and LFPQ). After the baseline assessment, participants engaged in either a moderate-to-vigorous intensity condition (HI) or a light intensity condition (LI) in a randomised, crossover design. The current study did not include a non-active control condition because we noticed high drop-out rate and incompliance of study protocols from participants during pilot testing, wherein participants were asked to complete 4 trials (preliminary, HI, LI, control). Further, previous studies comparing different exercise intensities to control condition revealed no difference in post-exercise total energy intake [23, 24]. As such, we decided to discard the control condition to ensure that the objective of the study (e.g. exploring intensity-specific differences in food reward and appetite sensation) could be tested with lower attrition rate.

**Fig. 1:**
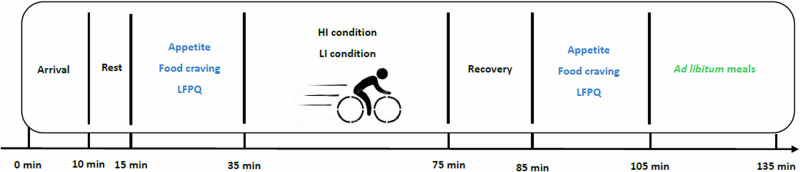
*HI* moderate-to-vigorous condition, *LI* light-intensity condition, *LFPQ* Leeds Food Preference Questionnaire. Diagram of experimental procedure.

The HI condition included a 40-min bout of exercise on a cycle ergometer (Monark Ergomedic 874E) at an intensity of 50% of the individuals PPO; during the LI condition, participants exercised at an intensity corresponding to 20% of individuals PPO for 40 min (Table 2 summarises exercise-related measures during HI and LI). A 40-min bout of exercise was chosen based on published research demonstrating modulatory effects of aerobic exercise on appetite sensation and food reward [11, 25], as well as on our pilot study indicating that this duration of exercise was tolerable by physically inactive adults. During both interventions, participants watched an emotionally neutral educational video (i.e. minutes 1–40 and 41–80 from *Wonder of the Universe*) that was not related to either exercise or nutrition. After the completion of exercise, participants had a seated rest for 10 min and completed the same measures again (i.e. VAS, FCQ-S, LFPQ). Afterward, participants were given a maximum of 30 min to have *ad libitum* meals (see Supplementary 1 for details) [13] and they could finish early if they wanted to. Figure 1 depicts the experimental procedure during the main trials.

**Table 2:** Summary of exercise related measures.

Condition	HR (bpm)	Estimated EE^a^ (kcal)	Power outputs (watts)	RPE	FS
HI	160.9 (15.4)	432.8 (101.0)	114.8 (31.4)	15.3 (2.0)	−0.7 (1.9)
LI	110.3 (11.6)	181.9 (60.8)	32.8 (9.0)	10.0 (1.8)	2.6 (1.9)

*HI* moderate-to-vigorous intensity condition, *LI* light intensity condition, *HR* heart rates, *bpm* beats per minute, *EE* energy expenditure, *RPE* ratings of perceived exertion, *FS* feeling scale.

^a^Estimated energy expenditure was calculated by the formula from Keytel et al. [38], where estimated EE for men = time of exercise × (0.6309 × mean HR during exercise + 0.1988 × weight + 0.2017 × age − 55.0969)/4.184 and for women = time of exercise × (0.4472 × mean HR during exercise − 0.1263 × weight + 0.074 × age − 20.4022)/4.184

All statistical analyses were performed using JASP version 0.18.1.0 (University of Amsterdam, The Netherlands), with a family-wise alpha of 0.05 set as the significance criteria. Missing data were imputed by mean values across all participants. Normality of data was first confirmed with Shapiro–Wilk test. Data of FCQ-S and fat consumption were log-transformed given the data were not normally distributed based on results of Shapiro–Wilk test. For changes in LFPQ outcomes (i.e. explicit wanting, explicit liking, implicit wanting), *Condition* (HI condition, LI condition) × *Time* (pre-test, post-test) × *Food bias* (fat bias, taste bias) repeated measured analysis of variances (RM ANOVAs) were performed. For appetite scores and state food cravings, *Condition* × *Time* RM ANOVA was conducted. For energy intake (i.e. macronutrient consumption, absolute and relative energy intake), paired-sample *t*-tests, with *Condition* as within-subjects factor, were performed. Greenhouse–Geisser correction was utilised if the assumption of sphericity was violated. Post hoc comparisons were corrected with Bonferroni-corrected *t*-tests. Partial eta square (*η*^2^_p_) effect sizes were reported in addition to significance testing, with *η*^2^_p_ of 0.01, 0.06, and 0.14 indicating small, medium, and large effect sizes, respectively [26]. Further, Cohen’s d with 95% confidence intervals (CI) was computed as effect size in post hoc comparisons, using appropriate variance corrections for repeated-measures comparisons (Cohen’s d_rm_) [27].

## Results

**Fig. 2:**
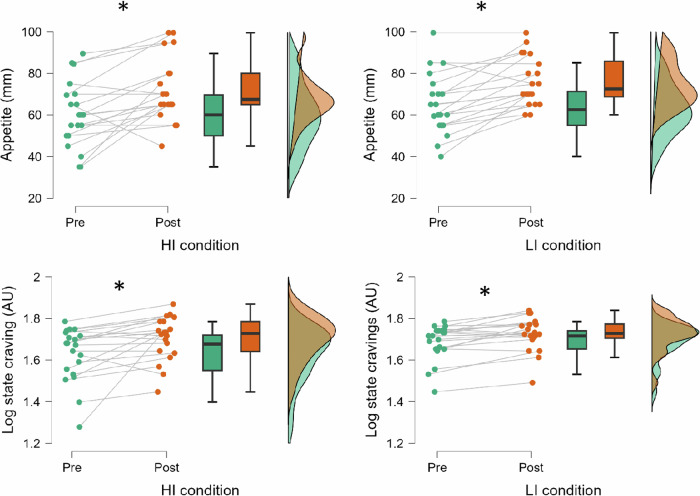
Appetite and food cravings responses to the moderate-to-vigorous intensity (HI) and light intensity (LI) conditions. AU arbitrary unit.

For appetite scores, analyses indicated a significant main effect of *Time* [*F*(1, 19) = 28.5, *p* < .001, *η*^2^_p_ = 0.60] with appetite scores increased from pre-test (62.7 ± 3.0) to post-test (74.1 ± 2.4). There was no *Condition* × *Time* interaction or main effect of *Condition* [*F*’s(1, 19) = < 2.3, *p*’s > 0.14 *η*^2^_p_ < 0.11]. Similarly, only a significant main effect of *Time* was found for log state food cravings [*F*(1, 19) = 14.7, *p* = 0.001, *η*^2^_p_ = 0.44] with higher state food cravings in post-test (1.7 ± 0.0) relative to pre-test (1.7 ± 0.0), regardless of exercise intensity. Figure 2 demonstrates changes in appetite and state food cravings.

**Fig. 3:**
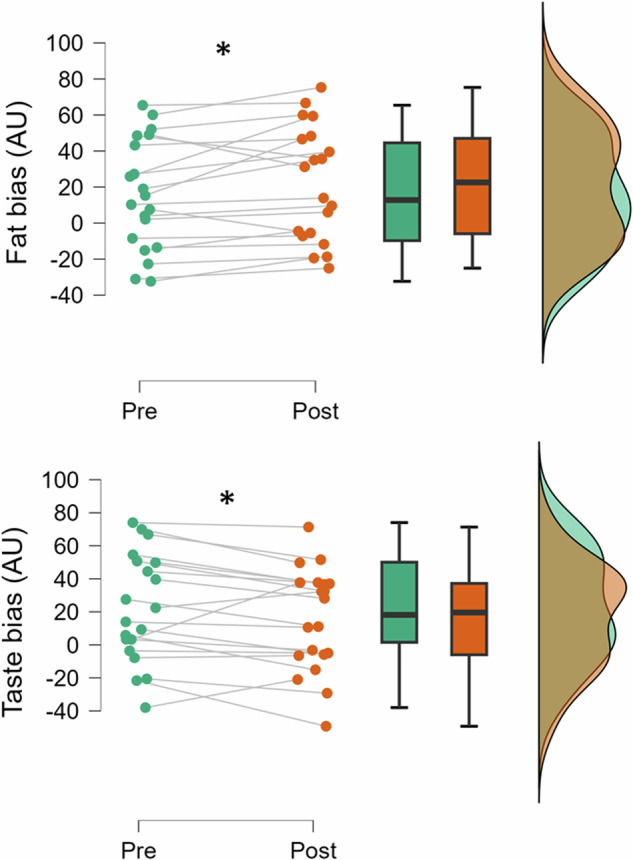
Modulations in implicit wanting as a function of time. Data were collapsed by experimental conditions. AU arbitrary unit.

For explicit wanting, analyses revealed no significant interactions or main effects, *F*’s(1, 19) < 1.6, *p*’s > 0.23, *η*^2^_p_’s < 0.08. Similarly, analyses revealed no significant interactions or main effects for explicit liking, *F*’s(1, 19) < 3.3, *p*’s > 0.09, *η*^2^_p_’s < 0.15. For implicit wanting, analyses showed a *Time* × *Bias* interaction, *F*(1, 19) = 21.5, *p* < .001, *η*^2^_p_ = 0.53. Post-hoc comparison indicated that fat bias increased from pre-test (15.3 ± 30.6) to post-test (21.8 ± 30.8), *t*(19) = 2.2, *p* = 0.04, d_rm_ = 0.50, 95% CI: 0.03, 0.96, whereas taste bias decreased over time (pre-test: 22.2 ± 32.9 vs. post-test: 15.1 ± 30.2), *t*(19) = −2.2, *p* = .04, d_rm_ = −0.49, 95% CI: −0.95, −0.02. Figure 3 shows modulations in implicit wanting as a function of experimental manipulation.

**Fig. 4:**
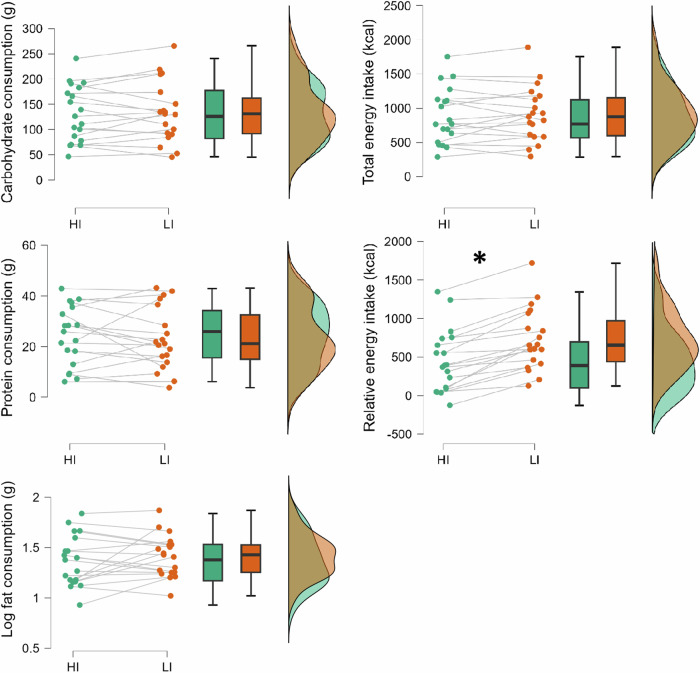
Food consumption and energy intake between moderate-to-vigorous intensity condition (HI) and light intensity condition (LI).

Energy intake data reported herein are from 19 participants due to food allergy from one participant. Table 3 summarises the energy intake following both HI and LI. There were no differences between post-HI *versus* post-LI on carbohydrate intake (HI: 131.4 ± 55.5 g vs. LI: 131.4 ± 55.5 g g), log fat intake (log HI: 1.4 ± 0.3 g vs. log LI: 1.4 ± 0.2 g), protein intake (HI: 24.3 ± 11.7 g vs. LI: 23.0 ± 12.3 g), and absolute energy intake (HI: 885.6 ± 406.9 kcal vs. LI: 909.4 ± 403.2 kcal). Relative energy intake was lower post-HI (451.8 ± 406.1 kcal) as compared to post-LI (729.1 ± 404.7 kcal), *t*(18) = −6.8, *p* < 0.001, d_rm_ = −1.56, 95% CI: −2.23, −0.88. Figure 4 summarises differences in energy intake between experimental conditions.

**Table 3:** Energy intakes following different exercise intensities.

Condition	Fat (g)	% energy intake from fat^*^	Carbohydrate (g)	% energy intake from carbohydrate^*^	Protein (g)	% energy intake from protein^a^	Total intake (kcal)	Relative intake (kcal)
HI	27.4 ± 16.7	27.7 ± 7.7	131.4 ± 55.5	60.6 ± 7.8	24.3 ± 11.7	11.0 ± 3.2	885.6 ± 406.9	451.8 ± 406.1
LI	29.0 ± 15.2	28.9 ± 5.2	131.4 ± 55.5	58.8 ± 4.4	23.0 ± 12.3	10.1 ± 3.7	909.4 ± 403.2	729.1 ± 404.7

*HI* moderate-to-vigorous intensity condition, *LI* light intensity condition.

^a^Energy intakes were calculated based on the following energy values: 1 g of fat = 9 kcal, 1 g of carbohydrate = 4 kcal, 1 g of protein = 4 kcal.

## Discussion

The main findings of the present study were that both moderate-to-vigorous and light-intensity aerobic exercise led to increased subjective appetite and cravings for food in physically inactive adults, and these alterations are underscored by enhanced preference towards high-fat and savoury foods. Furthermore, we observed similar absolute energy intake between HI and LI trials and less relative energy intake in the HI trial relative to LI trial.

Novelty of our findings lies on the modulations in processes of food reward in response to different intensities of exercise. Our fat and taste appeal bias scores indicated that acute bouts of 40-min aerobic exercise, regardless of intensity, increase implicit wanting for high-fat relative to low-fat foods and for savoury relative to sweet foods, and these alterations are accompanied by enhanced subjective appetite and food cravings post-exercise. Research regarding acute exercise effects on liking and wanting processes of food reward remain inconsistent across studies and such inconsistencies could be driven by heterogeneity in sex distribution and feeding state of participants [8–12]. The current study, therefore, is the first to investigate the acute exercise-induced alterations in food reward under the same feeding state (i.e. 4 h of fasting before each trial) in a sample consisting of both men and women. Importantly, we addressed this topic in a group of participants who were physically inactive and with lower fitness levels (see Table 1 for fitness profiles of participants).

Unlike previous studies with healthy adults who found either decreased implicit and explicit wanting [8–10] or unaltered liking and wanting for high-fat foods following acute aerobic exercise [11, 12], our data indicated increased implicit wanting toward high-fat foods regardless of exercise intensity. Regarding sweet/taste bias, our data showed increased motivation toward savoury foods following acute aerobic exercise regardless of intensity, which is inconsistent with studies that found either increased implicit wanting for high-sweet foods [8] or unchanged sweet/taste bias following acute aerobic exercise in healthy adults [9–12]. Discrepancies in processes of food reward between the current study and previous studies may be partially explained by differences in habitual physical activity and fitness levels of participants. Specifically, all of our participants were physically inactive and could be classified as lower-fit (mean estimated VO_2max_ was 39.1 ml kg min^−1^ and 32.8 ml kg min^−1^ for men and women, respectively). Notably, research has shown that individuals with lower physical activity or fitness levels tend to have increased disordered eating traits [28–30], as well as weaker satiety signalling and greater responsiveness to hedonic inputs [1]. Indeed, our participants demonstrated higher uncontrolled and emotional eating scores (22.8 and 9.8 for uncontrolled and emotional eating, respectively) as compared to scores (17.9 and 6.6 for uncontrolled and emotional eating, respectively) from 645 adults without disordered eating traits [31]. This implies that our participants may potentially possess elevated disordered eating traits. Therefore, in a group of physically inactive and lower-fit adults, the dose of aerobic exercise delivered in our study (i.e. 40 min at either light or moderate-to-vigorous intensity) might not be sufficient to increase satiety signalling and decrease responsiveness to hedonic food cues, resulting in increased appeal for high-fat and savoury foods. Our findings can be partially supported by recent studies with individuals with dysfunctions in the reward/motivation system (e.g. individuals with Methamphetamine dependence) who found that a 35-min bout of moderate-to-vigorous intensity aerobic exercise led to enhanced implicit wanting for high-fat savoury foods [32, 33]. Further research is needed to better clarify how individual differences in fitness and physical activity levels may alter food reward responses to acute bouts of aerobic exercise.

Furthermore, our data suggest that, despite greater energy expenditure during exercise, moderate-to-vigorous intensity exercise does not induce additional energy intake in post-exercise *ad libitum* meal compared to light-intensity exercise, which can be reflected by lower relative energy intake during HI trial relative to LI trial. This finding is relevant and can also be added to the rather scarce body of literature regarding intensity-dependent effects of acute exercise on eating behaviour. Interestingly, earlier studies focused on either adults with obesity [34] or healthy women [24] indicated similar appetitive responses and energy intake following high-intensity interval exercise and moderate-to-vigorous aerobic exercise. In contrast, a recent study in adults with obesity indicated that exercise duration alters subsequent energy intake and eating behaviour [25]. These findings, altogether, imply that exercise duration, but not intensity, might be a stronger modifier to the effects of acute aerobic exercise on subsequent eating behaviour.

Our data, collectively, may have the following practical implications: First, our results suggested that 40 min of aerobic exercise at either moderate-to-vigorous or light intensity was insufficient to exert appetite-reducing effect in healthy albeit inactive adults, suggesting alternative exercise protocols might be worth considering. Second, despite increased appetite, reduced relative energy intake in relation to moderate-to-vigorous exercise could still be relevant for the prevention of weight gain and metabolic disease, especially in inactive and/or lower-fit individuals who are more likely to gain weight and have higher risks to develop metabolic diseases [35]. Increased exercise intensity may also provide greater cardiovascular stimulation, potentially leading to improved fitness following prolonged training [36]. While both moderate-to-vigorous and light-intensity aerobic exercise may lead to increased appetite, moderate-to-vigorous intensity might still be preferable over light-intensity due to lower relative energy intake and greater health-related benefits.

Several limitations should be acknowledged. First, the lack of a non-exercise control condition may have introduced placebo and/or time effects to our food reward and appetite sensation data and our findings should, therefore, be interpreted with caution. For example, it is plausible that participants had increased appetite and food cravings due to passage of time, rather than due to exercise. However, we would like to argue that these risks, if any, were comparable between the HI and LI conditions and our data are novel in investigating into the intensity-specific differences in processes of food reward and appetite sensation in physically inactive and lower-fit adults. Relatedly, one may also argue that whether exercise led to changes in energy intake remains unclear in our data due to the lack of control conditions. We recognised this limitation but would like to highlight that some previous studies showed no difference between exercise condition and control condition in absolute energy intake [23, 24] and the intensity-specific difference in relative energy intake is another novelty of our study. Second, subjective question was employed to assess participants’ physical activity levels; however, this method may be subjected to limitations, such as inaccurate recall. To reduce the potential bias, future research may consider using objective wearable devices. Third, our participants started their main trials at either 10 a.m. or 3/4 p.m. (the same participant always started at the same time of the day for both trials). It is plausible that our results were confounded by inter-person differences in time of trials. Furthermore, a recent study indicated that early and late chronotype, as well as diurnal exercise timing (i.e. exercise in the morning versus exercise in the afternoon), may interactively affect appetite and food reward response to acute bouts of exercise [37]. Future studies should take these factors into consideration. Fourth, participants’ body weight and BMI were measured only during the preliminary trial. However, given that all participants were instructed to maintain their daily routine throughout the study period (i.e. energy intake and physical activity), confounding effect from potential weight change was mitigated in our study. Finally, our study design only allowed us to capture appetitive responses and eating behaviour within the first hour following exercise. Future research with prolonged post-exercise assessment may provide clearer picture of exercise intensity effects on appetitive responses, food reward, and eating behaviour.

In conclusion, the current study showed that, in physically inactive adults, acute bouts of aerobic exercise at both moderate-to-vigorous and light intensities may lead to increased appetite sensation and cravings for foods, which can be underscored by increased motivation to consume high-fat (relative to low-fat) and savoury (relative to sweet) tasting foods. Furthermore, despite greater energy expenditure during exercise, moderate-to-vigorous intensity exercise does not induce additional energy intake in post-exercise *ad libitum* meal compared to light-intensity exercise. Such findings may have relevant implications for weight management and cardiovascular health in physically inactive and/or lower-fit individuals.

## Supplementary information

Supplement 1

## Data Availability

Data will be made available on request.
