# Supplementary material for: Moderate-to-vigorous and light-intensity aerobic exercise yield similar effects on food reward, appetitive responses, and energy intake in physically inactive adults
Source: Eur J Clin Nutr. 2025 Feb 28;79(12):1204–10. doi: 10.1038/s41430-025-01574-5 (PMC12678172; doi:10.1038/s41430-025-01574-5)
Supplement: Supplementary file 1 — Supplement 1 [file 41430_2025_1574_MOESM1_ESM.docx]

**Supplement 1**

**Power estimation**

Using G*Power 3.1.9.7, the final sample (n = 20) was satisfactory to detect the effects of acute aerobic exercise on food reward measures (Cohen’s *d* = 0.67) ^1^ in a within-subjects, pre-post design (statistical power of 0.80 and *p* of .05 assumed).

**Protocols to measure aerobic capacity and estimated VO_2max_**

During the maximal aerobic capacity test, participants exercised at a constant speed equivalent to a peddling rate of 50 repetitions per minute (RPM) on a cycle ergometer (Monark Ergomedic 874E). The test protocol commenced at an exercise intensity equivalent to 70 watts, and the workloads increased by 30 watts every 3 minutes until participants reported voluntary exhaustion. A Polar HR monitor (Model H10; Polar Electro, Finland) was used to measure HR throughout the test. Ratings of perceived exertion (RPE) were assessed every 3 minutes with the Borg scale ranging from 6-20 ^2^. Two of the following criteria needed to be met to evidence maximal effort: (1) participants voluntarily reported exhaustion; (2) a peak heart rate ≥185 beats per minute ^3^ or a HR plateau ^4^; (3) RPE ≥17 ^2^. Individual PPO was determined by the following equation: (Watts_final_ + Time_final_/180) x 30, where Watts_final_ was power output in the final stage, Time_final_ was the time (in seconds) spent in the final stage, and 180 and 30 represent the duration of each stage (in seconds) and the increment of workload (in watts) per stage ^5^. PPO and weights (kg) were then used to estimate individual VO_2max_ following the equation by Hawley and Noakes (1992): VO_2max_ = (0.01141 x PPO + 0.435)/weight (kg).

**Description of psychometric questionnaires**

The Three Factor Eating Questionnaire-Short Form (TFEQ-r18) ^6^ measures three subscales of eating behaviour: cognitive restraint, uncontrolled eating, and emotional eating. Participants responded to 18 items of 4-point questions. Scores range from 0 to 100 for each subscale, with higher scores indicating a greater presence of problematic eating behaviour. The Food Craving Questionnaire-Trait-reduced form (FCQ-T-r) ^7^ assesses lack of control overeating, emotions experienced before or during food craving and consumption, and guilt from cravings and/or giving in to cravings. Participants responded to 15 items on a 6-point scale, with total scores ranging from 15 to 90. A higher score suggests more frequent habitual cravings, with a total score greater than 50 highlighting clinically relevant trait cravings ^8^. The Eating Disorder Examination Questionnaire (EDE-Q) ^9^ is a 28 items self-report questionnaire of eating disorder-associated symptoms with a 7-point scale of 0–6, and a severity screener of the last 28 days. Items 13 to 18 – on behavioural frequency rather than severity – were excluded from the data analysis. Twenty-two items in the EDE-Q constitute four subscales: weight concern, shape concern, eating concern, and restraint. The scores for relevant items under each subscale were added together and divided by the number of items to obtain the subscale scores. The mean total subscale scores signify the global score. Lastly, the Centre for Epidemiological Studies Depression Scale (CES-D-10) is a 10-item Likert scale questionnaire assessing depressive symptoms in the past week ^10^. It includes three items on depressed affect, five items on somatic symptoms, and two on positive affect. Options for each item range from “*rarely or none of the time*” (score of 0) to “*all of the time*” (score of 3). Total scores can range from 0 to 30. Higher scores suggest greater severity of symptoms.

**Appetite ratings**

To measure subjective ratings of appetite, 100 mm VAS were used to determine hunger (“*How hungry do you feel right now?*”), fullness (“*How full do you feel right now?*”), prospective consumption (“*How much food could you eat right now?*”), and the desire to eat (“*How strong is your desire to eat right now?*”) ^11^. Scores range from 0 to 100 mm, with higher scores indicating greater prevalence of the appetite measure. These VAS are sensitive to experimental manipulation and considered a reliable and valid measure of subjective appetite ^12^. A composite appetite score was derived from hunger and fullness scores using an approach recommended by Rogers and Hardman ^13^: (Hunger + (100-Fullness))/2.

**State food cravings**

In-the-moment food cravings were measured using the FCQ-S ^14^. Across 15 items, participants’ desire to eat, craving for food and emotional responses to food and consumption are measured. Participants respond to each item on a 5-point scale, where 1 corresponds with “*Strongly disagree*” and 5 corresponds with “*Strongly agree*”. These corresponding numbers are totalled to provide a score between 15 and 75, with a higher score indicating greater momentary craving.

**Food reward processes**

The LFPQ ^15,16^ is a validated computer-based assessment of the hedonic preference for food and measures explicit liking and implicit and explicit wanting as components of reward. Explicit liking is defined as the subjective pleasure elicited by the taste of food, whereas implicit wanting is the implicit motivational component of reward while explicit wanting refers to the subjective desire or craving for foods ^17^. The task uses a standardised set of 16 images depicting ready-to-eat foods that are common in the diet (see the table below for details). These images illustrate items that are either high (>40% energy) or low (<20% energy) in fat and either sweet or savoury taste and can be split into four categories; high-fat savoury (HFSA), high-fat sweet (HFSW), low-fat savoury (LFSA), and low-fat sweet (LFSW). Food reward is assessed according to the fat content and taste of these foods, which are comparable in protein content, palatability, and familiarity ^18^. The LFPQ incorporates two tasks where food items are either displayed in pairs (forced-choice task) or individually (single-food task). The forced-choice task measures the implicit wanting for foods and involves participants choosing the food they most want to consume “right now” from two items presented on the computer screen. A frequency-weighted algorithm is used to provide a score for implicit wanting, which combines reaction times with the frequency of choosing or avoiding a food ^16^. In the single-food task, participants are presented with each of the 16 food items and asked to rate “*How much do you want some of this food right now?*” and “*How pleasant would it be to taste some of this food right now?*”. Participants respond to each question on a 100-unit VAS that measure explicit wanting and liking, respectively. Fat appeal bias (FAB) and taste appeal bias (TAB) scores are additionally calculated for explicit liking, explicit wanting, and implicit wanting. Bias scores are calculated by subtracting mean scores across food groups (e.g., mean low-fat scores are subtracted from mean high-fat scores).

| Standardised food images used in LFPQ | | | |
| --- | --- | --- | --- |
| HFSA | HFSW | LFSA | LFSW |
| Garlic bread | Chocolate biscuits | Green salad | Mixed berry salad |
| Fries | Glazed doughnut | Broccoli | Skittles |
| Crips | Blueberry muffin | Vegetable rice | Haribo |
| Sausage | Milk chocolate | Bread roll | Banana |
| HFSA, high-fat savoury; HFSW, high-fat sweet; LFSA, low-fat savoury; LFSW, low-fat sweet. | | | |

**Ad libitum meals**

The *ad libitum* buffet meals were set up identically for each trial and consisted of corn flakes, white bread, whole wheat bread, semi skimmed milk, orange juice, cheese, ham/tuna (participants who did not eat pork had tuna instead), butter, salted crisps, salted pretzels, chocolate bars, butter shortbreads, biscuits, strawberry jam, apple, banana, peanut butter, mustard, mayonnaise, ketchup. All food was pre-weighed and presented in excess of expected consumption. Participants were told to eat until satisfied and that additional food was available if required. The buffet meal was consumed in isolation with no distraction and the use of computers or mobile phones was prohibited to minimise any influence on food consumption. At the end of the buffet meal, leftover food was weighed, and absolute and relative energy intake (kcal; energy intake during *ad libitum* meals – energy expenditure during exercise) and macronutrient composition of the food consumed (grams) was determined by calculating the weighted difference of each food item before and after each meal.

1 Yamada Y, Hiratsu A, Thivel D, Beaulieu K, Finlayson G, Nagayama C *et al.* Reward for fat and sweet dimensions of food are altered by an acute bout of running in healthy young men. *Appetite* 2024; **200**. doi:10.1016/j.appet.2024.107562.

2 Williams N. The Borg Rating of Perceived Exertion (RPE) scale. *Occup Med (Chic Ill)* 2017; **67**: 404–405.

3 American College of Sports Medicine. *ACSM’s guidelines for exercise testing and prescription*. Lippincott Williams & Wilkins: Philadelphia, 2018.

4 Freedson PS, Goodman TL. Measurement of oxygen consumption. In: Freedson PS, & GTL (ed). *Pediatric laboratory exercise testing: Clinical guidelines*. Human Kinetics: Champaign, IL, 1993, pp 91–113.

5 Hawley JA, Noakes TD. Peak power output predicts maximal oxygen uptake and performance time in trained cyclists. Springer-Verlag, 1992.

6 Karlsson J, Persson L-O, Sjöström L, Sullivan M. Psychometric properties and factor structure of the Three-Factor Eating Questionnaire (TFEQ) in obese men and women. Results from the Swedish Obese Subjects (SOS) study. *Int J Obes* 2000; **24**: 1715–1725.

7 Meule A, Hermann T, KÃ¼bler A. A short version of the Food Cravings Questionnaireâ€”Trait: the FCQ-T-reduced. *Front Psychol* 2014; **5**. doi:10.3389/fpsyg.2014.00190.

8 Meule A. Food cravings in food addiction: exploring a potential cut-off value of the Food Cravings Questionnaire-Trait-reduced. *Eating and Weight Disorders - Studies on Anorexia, Bulimia and Obesity* 2018; **23**: 39–43.

9 Fairburn CG, Beglin SJ. Eating Disorder Examination Questionnaire (EDE-Q 6.0). In: *Cognitive behavior therapy and eating disorders*. 2008, pp 309–313.

10 Andresen EM, Malmgren JA, Carter WB, Patrick DL. Screening for depression in well older adults: evaluation of a short form of the CES-D (Center for Epidemiologic Studies Depression Scale). *Am J Prev Med* 1994; **10**: 77–84.

11 Blundell J, De Graaf C, Hulshof T, Jebb S, Livingstone B, Lluch A *et al.* Appetite control: methodological aspects of the evaluation of foods. *Obesity Reviews* 2010; **11**: 251–270.

12 Beechy L, Galpern J, Petrone A, Das SK. Assessment tools in obesity — Psychological measures, diet, activity, and body composition. *Physiol Behav* 2012; **107**: 154–171.

13 Rogers PJ, Hardman CA. Food reward. What it is and how to measure it. *Appetite* 2015; **90**: 1–15.

14 Cepeda-Benito A, Gleaves DH, Williams TL, Erath SA. The Development and Validation of the State and Trait Food-Cravings Questionnaires ANTONIO CEPEDA-I~ENITO. *Behav Ther* 2000; **31**: 151–173.

15 Finlayson G, King N, Blundell JE. Is it possible to dissociate ‘liking’ and ‘wanting’ for foods in humans? A novel experimental procedure. *Physiol Behav* 2007; **90**: 36–42.

16 Dalton M, Finlayson G. Psychobiological examination of liking and wanting for fat and sweet taste in trait binge eating females. *Physiol Behav* 2014; **136**: 128–134.

17 Finlayson G, Dalton M. Hedonics of Food Consumption: Are Food ‘Liking’ and ‘Wanting’ Viable Targets for Appetite Control in the Obese? *Curr Obes Rep* 2012; **1**: 42–49.

18 Oustric P, Thivel D, Dalton M, Beaulieu K, Gibbons C, Hopkins M *et al.* Measuring food preference and reward: Application and cross-cultural adaptation of the Leeds Food Preference Questionnaire in human experimental research. *Food Qual Prefer* 2020; **80**. doi:10.1016/j.foodqual.2019.103824.
